# Fruits and vegetables moderate lipid cardiovascular risk factor in hypertensive patients

**DOI:** 10.1186/1476-511X-5-14

**Published:** 2006-06-05

**Authors:** Olugbenga Adebawo, Bamidele Salau, Esther Ezima, Olamilekan Oyefuga, Emmanuel Ajani, Gbolahan Idowu, Adekunle Famodu, Odutola Osilesi

**Affiliations:** 1 Department of Biochemistry, Faculty of Basic Medical Sciences, Obafemi Awolowo College of Health Sciences, Olabisi Onabanjo University, Remo Campus, Ikenne, Nigeria; 2 Department of Physiology, Faculty of Basic Medical Sciences, Obafemi Awolowo College of Health Sciences, Olabisi Onabanjo University, Remo Campus, Ikenne, Nigeria; 3 Department of Heamatology, School of Medicine, College of Medical Sciences, University of Benin, Benin-City, Nigeria

## Abstract

Hyperlipidemia is a major risk factor in etiology of cardiovascular disease. Previous studies have shown association between vegetarian diet and low total serum cholesterol as well as LDL-cholesterol which is a pointer to low risk of cardiovascular disease. Dietary fiber, antioxidants and other classes of nutrients have been reported to ameliorate cardiovascular risk factors. Fruits and vegetables being rich sources of fiber and antioxidants have been the focus in intervention studies. The current work reports the effect local fruits and vegetables on cardiovascular risk factors in African hypertensive subjects in an 8 week study. Though there was no significant difference in the Body Mass Index and HDL-cholesterol at the end of the eighth week, there were significant reductions (P < 0.05) in serum triglycerides (125.87 ± 6.0 to108.27 ± 5.49 mgdL^-1^); total serum cholesterol (226.60 ± 6.15 to 179.20 ± 5.78) and LDL-cholesterol (135.69 ± 5.56 to 93.07 ± 7.18 mgdL^-1^). We concluded that consumption of combination of local fruits and vegetables may reduce the incidence of cardiovascular risk factors in Africans.

## Introduction

Several epidemiological studies have implicated hypercholesterolemia and hypertriglyc eridemia as major factors in the etiology of vascular disease [1,2]. Other prospective studies have equally shown that serum cholesterol [3], LDL-cholesterol [4], body mass index or obesity index, atherogenic index [5] and coronary risk index [6] are major risk factors in cardiovascular diseases.

Some studies have investigated the nutritional status of white and black American vegetarians [7,8]. These authors reported lower serum total and low-density lipoprotein (LDL) as well as cholesterol in vegetarians and suggested lower cardiovascular risk and essentially lower blood pressure (BP)among vegetarians. Similar results were obtained with Native African vegetarians [9].

Hypertension is a common cause of cardiovascular disorders and is essentially associated with abnormal lipid and altered glucose metabolism [10,11]. Among the classes of food that have been taken to be beneficial in reducing risk of cardiovascular disorders are fruits and vegetables due to their high level of fibres [12], antioxidants [13] and complex carbohydrates [14].

Intervention studies of disease prevention with fruits or vegetables or both in Africans are rare. It is important to determine if fruits and vegetables are associated with lower BP and blood lipid and other risk factors for cardiovascular complications.

## Materials and methods

Twenty hypertensive patients were randomly selected from the cardiovascular clinic of Olabisi Onabanjo University Teaching Hospital (OOUTH). The subjects that had been on diabetic drugs for over one year were educated on the purpose of the research work and they all consented. The average nutrients intake by the subjects using estimated food records were calculated.

Two weeks after the first table contact with the subjects, blood specimen were taken and other parameters were measured for analysis to serve as baseline. Edible portion of fairly ripe fruits (banana, pawpaw, grape fruits, tangerine and pineapple) were diced mixed together in equal weight fruits salad with exception of banana which two fingers were given per serving. Two servings of fruits salad (each measuring 100 g) were given per day. Edible green leafy vegetable including fluted pumpkin leaf, spinach and waterleaf were diced and given in 100 g portion per day after moderate cooking. The supplementation of the normal diet of the hypertensive subjects with fruits and vegetables was carried out for eight weeks after which it was stopped for two weeks.

Out of twenty subjects, only fifteen faithfully complied with the feeding regime and this form the basis for the computation of results.

### Analytical method

After the baseline measurements, the blood specimens were taken and parameters measured at two weeks interval for a period of eight weeks. Blood Pressure values were extracted from the patients' case notes. Triglycerides, total serum cholesterol and high-density lipoprotein cholesterol (HDL cholesterol) concentration were determined by enzymatic method using analytical kits. (Randox Laboratories U.S.A). While low density lipoprotein (LDL cholesterol), was obtained by deduction. Atherogenic index (A.I) was calculated using the formula of Abot et al [15] and coronary risk index (C.R.L.) was obtained by the method of Alladi et al [16]. Body mass index was calculated using the method of Garrow and Webster. [17].

### Statistical analysis

The experimental design was completely randomized. The data were analyzed at 95% level of significance using the two-tale Student's test.

## Results

Table 1 shows estimated nutrient intake of subjects and the percentage difference between the baseline values and the values in the presence of fruits and vegetables. Reduction was noticed in intake of energy, sodium, fats and related compounds while increased was observed in all other nutrients.

**Table 1:** Estimated daily nutrient intake of hypertensive subjects

**Nutrient**	**Baseline**	**Fruit and vegetable supplemental diet**	**% Difference**
Energy (calories)	13448 ± 19.60	9818.98 ± 23.00	
Carbohydrate (g)	543.30 ± 21.30	617.22 ± 10.60	
Fat and related compound (g)	28.59 ± 5.00	20.32 ± 3.00	
Protein (g)	189.10 ± 6.90	200.03 ± 4.60	
Riboflavin (mg)	5.10 ± 1.60	6.04 ± 1.90	
Vitamin C (mg)	386.50 ± 12.70	1076.26 ± 35.00	
Soluble fibre (g)	20.20 ± 8.30	35.9 ± 4.609	
Insoluble fibre (g)	6.16 ± 1.70	30.56 ± 0.60	
Total fibre (g)	26.14 ± 8.10	64.49 ± 3.56	
Niacin (mg)	14.24 ± 3.50	23.75 ± 1.80	
Thiamine (mg)	2.38 ± 1.30	4.12 ± 0.70	
Vitamin A (Iu)	2996.90 ± 27.00	4531.78 ± 14.60	
Sodium (mg)	2256 ± 57.90	1608.43 ± 45.76	
Potasium (mg)	1826.10 ± 4130	3247.28 ± 12.90	
Calcium (mg)	1248.10 ± 46.90	1738.88 ± 26.50	
Phosphorus (mg)	1077.50 ± 23.90	2096.98 ± 36.20	
Iron (mg)	74.18 ± 1.40	81.09 ± 0.90	
Magnesium (mg)	97.40 ± 6.40	128.30 ± 1.30	

Table 2 shows Body mass index (BMI) and lipid profiles of the subjects. Significant differences (P < 0.05) were noticed between the baseline values and the eighth-week values for all the parameters except the BMI and HDL cholesterol. Also significant differences (P < 0.05) was noticed between the eighth week values and the tenth week values in Triacylgleride total serum cholesterol, however, no significance difference (P > 0.05) was noticed in BMI and HDL cholesterol.

**Table 2:** BMI and lipid profile of hypertensive subjects

	**Baseline**	**Week 2**	**Week 4**	**Week 6**	**Week 8**	**Week 10**
**BMI**	25.53 ± 1.44	25.64 ± 1.44	25.72 ± 1.44	25.88 ± 1.45	25.79 ± 1.46	25.83 ± 1.47
Triglycerides mgdL^-1^	125.87 ± 6.85	131.80 ± 7.61	131.07 ± 5.56	113.67 ± 6.54	118.27 ± 10.49	105.73 ± 5.75
Total serum Chol. mgd^-1^	226.60 ± 6.15	213.53 ± 8.35	199.80 ± 8.34	184.53 ± 4.04	179.20 ± 5.78	190.60 ± 4.7
HDL chol mgd^-1^	64.53 ± 2.40	63.20 ± 5.56	62.00 ± 17	58.73 ± 1.64	62.67 ± 1.64	60.27 ± 1.25
LDL chol mgdL^-1^	135.69 ± 5.56	125.09 ± 7.55	112.12 ± 5.86	102.25 ± 4.67	93.07 ± 7.18	109.01 ± 4.2

Table 3 shows a significant difference (P < 0.05) between the baseline values and the eighth-week values in Atherogenic index and Systolic blood pressure while no significant difference (P > 0.05) was between the eighth-week values and tenth-week values.

**Table 3:** Atherogenic index, coronary risk index and blood pressure of hypertensive subjects

	**Baseline**	**Week 2**	**Week 4**	**Week 6**	**Week 8**	**Week 10**
Atherogenic Index	2.34 ± 0.12	2.20 ± 0.14	1.92 ± 0.11	1.93 ± 0.14	1.79 ± 0.11	1.73 ± 0.13
Coronary Risk Index	3.90 ± 0.14	3.72 ± 0.15	3.11 ± 0.11	3.39 ± 0.15	3.21 ± 0.13	3.26 ± 0.13
Systolic mm Hg	155.33 ± 7.55	152.67 ± 6.36	151.33 ± 3.63	149.33 ± 6.21	141.343 ± 3.89	143.33 ± 4.33
Diastolic mm Hg	89.33 ± 3.44	90.67 ± 3.30	94.67 ± 3.22	86.00 ± 3.49	86.00 ± 3.49	88.00 ± 2.43

## Discussion

Serum cholesterol is a major causative agent in the development of coronary heart disease (CHD). Some studies have demonstrated a fall in total serum cholesterol resulting from ingestion of soluble fibre [19,20] suggesting that high cereal fiber may protect against ischemic heart disease (IHD) as well as high blood pressure, serum cholesterol and triglyceride levels. Our study with daily intake of ≥ 3 times of different types of fruit and vegetables totaling 500 g for 8 weeks produced significant reduction in systolic blood pressure. The reduction in the blood pressure might be due to significant high level of fibre. It has been shown that African local fruits and vegetables are rich in dietary fibre, which has been reported to have hypotensive and hypocholesterol effects [21]. Replacing animal products with vegetarian diets have shown reduction in blood pressure in normotensive as well as hypertensive individuals [22,23]

In a large randomized controlled trial of diet and blood pressure that provided a diet for 8 week that included 8.5 or 3.6 (control) servings of vegetables and fruit daily, the participants who consumed the higher vegetable and fruit diet had a greater reduction in systolic and diastolic than did the control subjects [24].

Elevated serum total cholesterol, LDL cholesterol concentration, are identified risk factors for coronary artery disease [25,26]. However, in this study, we observed lower serum total cholesterol, LDL cholesterol and triacylglycerol. These results may account for the significant reduction in BP in our patients. A recent study has reported similar observation with 900 effects of garlic may be due to inhibition of hepatic cholesterol biosynthesis [29].

In another study, [21,22] African local fruits and vegetables have been shown to be rich in dietary fibre, which have been reported to have hypotensive and hypocholestrolemic effects. The plant sterols have also been shown to produce a reduction of plasma low-density lipoprotein-cholesterol and produced prolonged platelet aggregation after collagen epinephrine activation (33).

Our results indicate that fruits and vegetables associated with lower cardiovascular risk factors; lower BP, cholesterol, triciglycerol and soluble fiber thus preventing premature cardiovascular disorders. We therefore conclude that consumption of a combination of fruits and vegetable may enhance healthier lifestyle resulting in the more favourable status of decreased incidence of cardiovascular risk factors.
